# Editorial: Xenobiotics, gut microbiota, and chronic disease

**DOI:** 10.3389/fnut.2026.1778844

**Published:** 2026-02-03

**Authors:** Bei Gao, Ying Zhang, Pengcheng Tu

**Affiliations:** 1 Jiangsu Key Laboratory of Atmospheric Environment Monitoring and Pollution Control, Collaborative Innovation Center of Atmospheric Environment and Equipment Technology, School of Environmental Science and Engineering, Nanjing University of Information Science and Technology, Nanjing, China; 2 BioMarin, San Rafael, CA, United States; 3 Department of Environmental Health, Zhejiang Provincial Center for Disease Control and Prevention, Hangzhou, China

**Keywords:** chronic disease, gut microbiota, live microbe intake, probiotics, xenobiotics

Mounting evidence underscores the essential role of gut microbiota in human health and disease, establishing it as a promising therapeutic target for various chronic conditions. The diversity and composition of the gut microbiota can be influenced by numerous factors—including xenobiotics. This Research Topic of *Frontiers in Nutrition*, titled “*Xenobiotics, Gut Microbiota, and Chronic Disease*,” presents eight original research articles and six reviews addressing chronic conditions such as coronary heart disease, depression, chronic obstructive pulmonary disease (COPD), liver disease, ulcerative colitis, functional dyspepsia, obesity, and renal disease.

The gut microbiota is closed related to chronic disease. Wang, Tan et al. conducted a meta-analysis linking small intestinal bacterial overgrowth to metabolic dysfunction-associated steatotic liver disease. Ren et al. reviewed gut microbiota's role in ulcerative colitis, emphasizing its therapeutic potential. Lei et al. combined Mendelian Randomization with experimental validation to establish a causal relationship between gut microbiota, protein metabolism, and non-alcoholic fatty liver disease. Lou et al. examined connections between dietary patterns, gut microbes, and obesity, proposing microbiota-targeted dietary interventions as a viable anti-obesity strategy.

Certain xenobiotics adversely affect the gut ecosystem. For example, exposure to toxic heavy metals can disrupt microbial balance, altering metabolism and physiological functions which linked to metabolic and other health disorders. Conversely, the gut microbiota can modulate how heavy metals are processed by acting as a physical barrier, affecting intestinal pH, oxidative balance, and the expression of detoxification-related enzymes or proteins (Zhu et al.). In another study, Gao *et al*. used a complete water replacement model to show that chronic cola intake, regardless of sugar content, significantly disrupts gut microbiota and impairs immune and renal function (Gao, Li et al.).

Several studies highlight beneficial effects of probiotics and dietary microbes. A cross-sectional National Health and Nutrition Examination Survey (NHANES) analysis found that dietary intake of live microbes is associated with higher serum levels of fat-soluble vitamins (Zheng et al.). A systematic review of eight randomized controlled trials reported that probiotics increase high-density lipoprotein cholesterol, glutathione, and total antioxidant capacity, while reducing low-density lipoprotein cholesterol, malondialdehyde, high-sensitivity C-reactive protein, toll-like receptor 4, and interleukin-6 (Yang et al.). A negative association was observed between a dietary index for gut microbiota and COPD (Ao et al.). In COPD patients aged ≥40 years, higher dietary live microbe intake was inversely associated with depression prevalence (Gao, Ling et al.). Data from NHANES (2011–2016) indicated that probiotic and yogurt consumption benefit hepatic steatosis (Song et al.). Medium-to-high live microbe intake was linked to better cardiovascular health (Life's Essential 8 scores) in U.S. adults (NHANES 2005–2018) (Wang et al.). Pretreatment with *Lactobacillus casei* strain Shirota mitigated renal injury severity, likely via anti-inflammatory mechanisms (Chan et al.). However, applying dietary microbial modulation in functional dyspepsia remains controversial and challenging (Huang et al.).

In summary, the research compiled in this Topic substantiates the pivotal role of gut microbiota in the pathogenesis of chronic diseases, and elucidates the profound impact of xenobiotic exposure on its composition and subsequent health outcomes. These works collectively reinforce the theoretical foundation of targeting the gut microbiota for therapeutic intervention, while providing actionable strategies for its modulation, ranging from dietary adjustments and probiotic supplementation to the mitigation of environmental toxicant exposure. The fundamental significance lies in expanding the perspective on chronic disease prevention and management from traditional organ pathology to a holistic “host-microbiota symbiosis” paradigm, centering on the microbial ecosystem. This shift offers a transformative framework for understanding disease complexity and developing novel interventions.

Looking ahead, the field must advance through three critical transitions to remain at the forefront. First, a shift from correlation to mechanism is imperative. Leveraging integrated multi-omics, organoid models, and targeted microbiota transplantation, future studies must delineate the precise molecular and cellular pathways underlying the “xenobiotic-microbiota-host” axis. Second, a move from generality to personalization is essential. Given the vast inter-individual variability in microbiome genetics, diet, and lifestyle, the integration of artificial intelligence with multidimensional data will be crucial to develop predictive models and enable truly tailored interventions. Ultimately, the transition from scientific insight to clinical and public health translation must be realized. This entails rigorously evaluating how microbiota-targeted strategies, such as next-generation probiotics, synbiotics, and precision nutrition, can be safely and effectively integrated into clinical guidelines and public health policies for chronic disease management, while assessing their long-term efficacy and risks. The knowledge synthesized in this Research Topic provides a critical foundation for ushering in this new era of precision medicine and nutrition, anchored in a deep understanding of the gut microbiota.

